# Perioperative antithrombotic medication: An approach for the primary care clinician

**DOI:** 10.4102/phcfm.v16i1.4555

**Published:** 2024-08-09

**Authors:** Daniël J. Laäs, Mergan Naidoo

**Affiliations:** 1 Department of Anaesthesia and Critical Care, Faculty of Health Science, University of KwaZulu-Natal, Durban, South Africa; 2 Department of Family Medicine, Faculty of Health Sciences, University of KwaZulu-Natal, Durban, South Africa

**Keywords:** perioperative, antithrombotics, anticogaulants, antiplatelets, neuraxial anaesthesia

## Abstract

The primary care clinician faces many challenges and is often left to manage complex pathology because of resource constraints at higher levels of care. One of these complex conditions is the perioperative management of antithrombotic medication. This narrative review is focused on helping the clinician navigate the complex path and multiple guidelines related to the perioperative use of antithrombotic medication. Perioperative antithrombotic guidelines (American College of Chest Physicians, European Society of Regional Anaesthesia, and American Society of Regional Anesthesia) and relevant publications were identified by a PubMed search using the terms perioperative AND anticoagulants OR antithrombotics AND guideline. Issues relevant to clinical practice were identified, and attempts were made to explain any ambiguity that arose. Adhering to basic pharmacological principles and evidence-based guidelines allows for the safe usage of antithrombotics. Knowing when to stop, continue, bridge and restart antithrombotic medication prevents perioperative morbidity and mortality. Stopping antithrombotic medication too early can lead to thromboembolic complications associated with their primary disease process. Not stopping antithrombotic medication or stopping it too late can potentially cause life-threatening bleeding, haematomas and increased transfusion requirements.

## Introduction

It is common for clinicians involved in perioperative care to encounter patients on antithrombotic medication. In 2017, over 4 million patients were prescribed an anticoagulant in the United States Medicare database.^1^ The number of inpatient and day-case surgeries is increasing yearly, and it is a case of when, rather than if, the primary care clinician encounters a patient on antithrombotic therapy presenting for elective or emergency surgery.^2,3^ Patients on antithrombotics are at increased risk of dying, cerebrovascular accidents (CVAs), gastrointestinal bleeds (GIB), permanent paralysis because of spinal haematomas, endovascular stent thrombosis, mechanical heart valve thrombosis, deep venous thrombosis (DVT) and the complications arising from embolisation.^4^ Knowing how to manage patients on these agents is essential in the primary care clinician’s armamentarium.

The pre-operative challenge is balancing the risk of bleeding from surgery and weighing it up against the risks associated with cessation of medication. Stopping antithrombotics carries a significant risk of thrombosis of coronary, carotid, intracerebral or other stents, intravascular devices and arterial and venous vessels.^5^ In the immediate postoperative period, a hypercoagulable state ensues, but it is also when a patient is at the highest risk of bleeding from a surgical wound.^6^ Reinitiating antithrombotic therapy must be performed promptly, but it must be balanced against the risk of haemorrhagic complications.

This review will attempt to guide the primary care clinician by summarising the most recent published guidelines on the topic. These include the American College of Chest Physicians (ACCP) Perioperative Management of Antithrombotic Therapy practice guidelines, European Society of Regional Anaesthesia (ESRA), Regional Anaesthesia in Patients on Antithrombotic Drugs guidelines and the American Society of Regional Anesthesia (ASRA) guidelines on Regional Anesthesia in the Patient Receiving Antithrombotic or Thrombolytic Therapy.^7,8,9^ The South African Society of Anaesthesiologists (SASA) published the Guidelines for Regional Anaesthesia in South Africa in 2016, but the section on perioperative anticoagulation and regional anaesthesia is based on ASRA guidelines; hence the ASRA guidelines will be discussed.^10^

## Methods

This narrative review addresses key issues highlighted in the ACCP, ESRA and ASRA guidelines. In addition, we did a focused search of PubMed using the following medical subject heading (MeSH) terms: Perioperative AND Anticoagulants OR antithrombotics AND guideline. The search was further refined by using only recent literature (5 years), meta-analysis, systematic reviews and guidelines. Only articles written in English were reviewed.

A total of 99 articles were included in the results. Abstracts were reviewed, and relevant full text was included in the review. Relevant local journals’ (South African Medical Journal, South African Journal of Anaesthesia and Analgesia) databases were also reviewed using the same MeSH terms and applicable articles were included.

American College of Chest Physicians recommendations were formulated using a series of patient, intervention, comparator and outcome (PICO) questions posed to a panel of experts. The panel sought the highest quality of available evidence and made recommendations based on a modified Delphi technique. The guidelines were generated by grading the Recommendations, Assessment, Development, and Evaluation (GRADE) methodology. Of the 44 recommendations, 33 were of very low certainty of evidence. This speaks to the relative paucity of information on the subject and is a confounder when applying these guidelines to clinical practice. However, given that these guidelines are written with a primary focus on safety, it is still reasonable to apply them. American Society of Regional Anesthesia and ESRA guidelines used a similar approach in synthesising and compiling the recommendations, but grading was performed differently. Similar limitations were found in all guidelines, as complications such as spinal haematomas are rare. The bulk of the recommendations are made from observational and epidemiological studies, with some being made from expert opinion.

## Pharmacology

**FIGURE 1:**
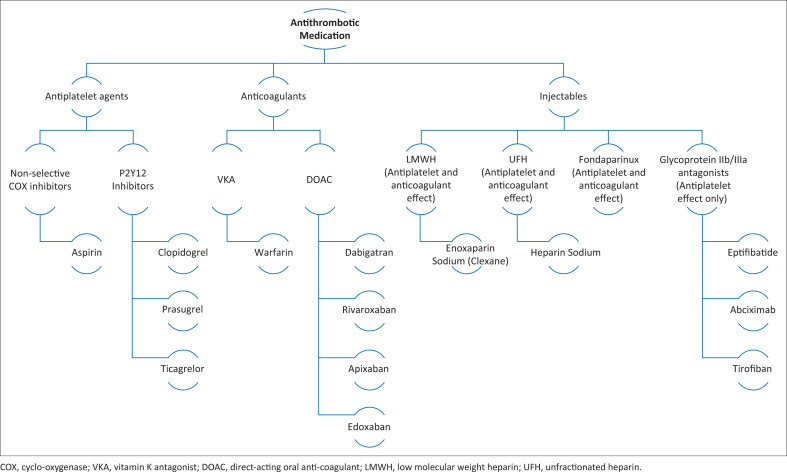
Classification of antithrombotic medication.

Agents that inhibit haemostasis are classified into either antiplatelets or anticoagulants (Figure 1). Injectable antithrombotics have dual effects. The antiplatelet medication inhibits platelet aggregation and thrombus formation. They are most useful at preventing arterial thrombi and indications for their use include treatment and prophylaxis against vascular occlusive disease states and prevention of stent thrombosis.^4^ Aspirin (ASA), a non-selective cyclo-oxygenase (COX) inhibitor, is used in secondary prophylaxis against cardiovascular disease, treatment of peripheral vascular disease and acute coronary syndromes (ACS) and as primary prophylaxis against stent thrombosis. The P2Y12 inhibitors (clopidogrel, ticlopidine, ticagrelor, prasugrel and cangrelor) are used in combination with ASA to treat ACS and prevent stent thrombosis. If not stopped preoperatively, P2Y12 inhibitors have a powerful antiplatelet effect and increase blood loss and the need for transfusions.^7,11^

Oral anticoagulants are used to prevent and treat venous thromboembolism (VTE). Vitamin K antagonists (VKAs) such as warfarin prevent mechanical heart valve thrombosis, embolic complications arising from valvular and non-valvular atrial fibrillation (AF) and recurrent deep vein thrombosis (DVT). Warfarin is also used to treat DVTs and pulmonary embolism.^12^ Regular international normalised ratio (INR) monitoring is required to assess the adequacy of warfarin therapy. An INR of two to three is desirable for most conditions, while an INR of 3–4 is recommended for mechanical valves at high risk of thrombosis.^13^

Direct-acting oral anticoagulants (DOACs) are a group of drugs that directly inhibit thrombin or factor Xa. Dabigatran is the classic direct thrombin inhibitor, and rivaroxaban, apixaban and edoxaban are factor Xa inhibitors. These agents are used for similar indications as warfarin but are considered superior as they do not require routine drug monitoring and have less bleeding risk. Direct-acting oral anticoagulants have three significant drawbacks: they cannot be used in patients with mechanical heart valves as they increase mortality because of thrombotic complications, emergent reversal of anticoagulation is challenging as antidotes are not as readily available as vitamin K is for warfarin and they are more expensive than vitamin K antagonists and not readily available in the public sector.^14^ The single exit price (SEP) of 30 tablets of Xeralto 10 (Bayer [Pty] Ltd) is R1130.39 compared to an SEP of R180.09 for 100 tablets of Cipla-Warfarin’s 5mg (Cipla Medpro [Pty] Ltd).^15,16^

Injectable antithrombotics include heparins (unfractionated and low molecular weight), fondaparinux (direct factor X inhibitor) and glycoprotein IIb/IIIa inhibitors. Heparins are used ubiquitously and will be discussed further. Unfractionated heparin (UFH) exerts its effects by binding to anti-thrombin III, which inactivates factors X and II (thrombin). Low molecular weight (LMWH) heparin exerts most of its effects directly on factor X. Enoxaparin sodium, better known by its trade name Clexane (Sanofi Aventis South Africa (Pty) Ltd) is the most commonly used LMWH in the public sector. Low Molecular weight heparin is more expensive than UFH (SEP R 784.02 per packet of 10 prefilled Clexane 40 mg injections vs. SEP R66.71 for X5, 5 mL vials of 5000 Iµ/mL UFH). However, given its predictable pharmacokinetics, safety of being prefilled and ease of administration, LMWH is the preferred injectable anticoagulant in clinical practice and is used extensively in the public sector in South Africa.^17,18^ It is administered through the intravenous or subcutaneous route, as a bolus. Protamine sulphate can fully reverse UFH’s anticoagulant effect, while LMWH can only be partially reversed with protamine (60%). Heparins are used as prophylaxis against thrombosis, as well as for treating thromboembolism and perioperatively to bridge patients who cannot take oral anticoagulants and are at high risk of thrombotic complications.

Fondaparinux is an alternative agent to classical heparins that can be used for heparin resistance or heparin-induced thrombocytopaenia. Its widespread use is mainly limited by its cost (SEP R2333.69 per X10 injections).^19^

## Perioperative considerations

Patients on antithrombotics present for elective or emergency surgery. Emergency surgery must proceed, and one must deal with the consequences of bleeding as best as possible. Elective surgical patients on antithrombotics are subdivided into three groups: high risk, low risk and intermediate risk of thromboembolism. The low- and intermediate-risk groups can be pooled together as perioperative management of antithrombotics is similar.^7^ Patients are deemed to be at high risk if they have a greater than 10% chance of developing VTE. Three patient populations form the high-risk category. The first group of patients has a known diagnosis of AF and a CHA_2_DS_2_VASc score greater than seven or less but with recent CVA or rheumatic heart disease.^7^ A subset of patients with mechanical heart valves forms the second group. The high-risk groups are those with mitral valve prosthesis and an associated major risk factor for stroke, aortic and mitral valves with a tilting disc or cage ball mechanism and recent stroke. The final group of patients are those with hypercoagulable conditions. Examples include VTE within the previous 3 months, severe thrombophilia or antiphospholipid syndrome.

In addition to the risk of VTE, the procedure can be classified as having high, intermediate and low risk of associated bleeding. Surgeries with 30-day bleeding risk greater than 2% are deemed as high bleeding-risk surgeries. Such procedures include major cancer surgery, thoracic surgery, cardiac surgery, vascular surgery and intracranial surgery. Any patient who underwent a neuraxial (spinal) anaesthetic is also deemed to be at high risk of bleeding because of the potential of permanent paralysis associated with spinal haematomas. Minor orthopaedic surgery (arthroscopy) and abdominal and gynaecological surgery are intermediate bleeding risk surgery examples.^20^ Low-bleeding risk surgery includes minor dermatological procedures, cataract procedures, minor dental procedures and pacemaker implantation.^20^ The most important patient groups to identify are those at high risk of bleeding and thrombosis. Following the correct classification, perioperative management largely depends on the antithrombotic agent administered.

## Antiplatelet agents

Antiplatelet agents prevent coronary, cerebral and peripheral vascular events. Patients with vascular risk factors are at high risk of perioperative myocardial adverse events (cardiac death, non-fatal myocardial infarction and cardiac arrest).^21^ Prematurely or incorrectly stopping medication in vasculopathy is potentially fatal. European Society of Regional Anaesthesia guidelines recommend that ASA at doses less than 200 mg can be continued in patients undergoing non-neurosurgical or ophthalmological procedures with a minimal increase in bleeding events.^9,18^ The same recommendation does not apply to the P2Y12 inhibitors and they should be stopped routinely before elective surgery. Prasugrel therapy must be stopped 7–10 days prior to surgery, clopidogrel 5–7 days and ticagrelor 3–5 days. P2Y12 inhibitors can be recommenced within 24 h after surgery, as it takes 4–5 days for them to reach a therapeutic effect when administered without a loading dose.^7^

Patients with a recently placed coronary artery stent should have all non-emergent surgery delayed for at least 6 weeks to 3 months as they require dual antiplatelet therapy (DAPT). In the elective setting, DAPT should be continued for 6–12 months, depending on the type of stent. Emergency surgery should proceed knowing that the patient has an elevated bleeding risk.^7^ Should the patient require elective surgery outside of the critical 6 weeks to 3 months period but still within the recommended DAPT period, the following approach is recommended:

Preoperatively, ASA should be continued and P2Y12 inhibitor stopped. The P2Y12 inhibitor can be reinitiated as soon as the bleeding risk after surgery allows; ideally, this should occur within the first 24 h.^7^

Cangrelor is used where bridging of a P2Y12 inhibitor is deemed essential. The drug must be initiated within 72 h of stopping the P2Y12 inhibitor and stopped 1–6 h prior to surgery. It can be reinitiated 4–6 h after surgery.

Bridging therapy with cangrelor or a heparinoid is recommended only if a stent has been placed in a critical area, such as the left main stem or it has been placed within the prior 3 months.^7^ Minor ophthalmological, dermatological and dental procedures can continue without DAPT interruption with no increased risk of bleeding.^7^

### Vitamin K antagonists

Vitamin K antagonists should be stopped at least 5 days prior to surgery. Routine monitoring of INR is not indicated after cessation of VKAs unless the patient is known to have a labile INR. Medication should be restarted within 24 h after surgery unless there is ongoing bleeding, expected additional intervention or a clinical reason to withhold. Full anticoagulation will only occur after 4–8 days. This recommendation applies to all elective surgery, and bridging anticoagulation, usually in LMWH or UFH, is only required for high-risk patients. Simple dental extractions, dermatological procedures and cataract removal are the exception and do not require cessation of anticoagulation.^7^

In patients presenting for emergency surgery with an elevated INR and at high risk of bleeding, the following strategy is recommended:

1. Stop warfarin
2. Administer intravenous vitamin K 10 mg if actively bleeding
3. Administer prothrombin complex 25 IU/kg – 50 IU/kg OR
4. Fresh Frozen Plasma 10 mL/kg – 20 mL/kg
5. Monitor INR every 30 min until goal INR has been achieved.^22,23^

### Direct oral anticoagulants

Direct oral anticoagulants confer a significant advantage over VKAs because they have a predictable pharmacological onset and offset time. The anti-factor Xa agents should be stopped 2 days before major surgery, and dabigatran should be stopped 2 days before elective surgery in a patient with a creatinine clearance (CrCl) >50 mL/min and 4 days prior to surgery in those with a CrCl < 50 mL/min. In low bleeding-risk procedures, all DOACs can be resumed 24 h after surgery, while in high bleeding-risk procedures, DOACs should be resumed 48–72 h after surgery. Neither routine perioperative drug monitoring nor heparin bridging is recommended because of the favourable pharmacokinetics and the rapidity (2 h) in which DOACs reach peak effect.^7^

### Bridging and heparins

The decision to continue or withhold anticoagulants or bridge the patient with heparin is based on three principles: the type of oral anticoagulant given, bleeding risk of surgery and risk of thrombosis. Routine heparin bridging after cessation of VKAs is not recommended as it increases bleeding risk without conferring additional protection against thrombosis.

The exception to this, where bridging therapy is reasonable, is in patients at high risk of thrombosis, i.e., patients in whom VKAs have been stopped.

**FIGURE 2:**
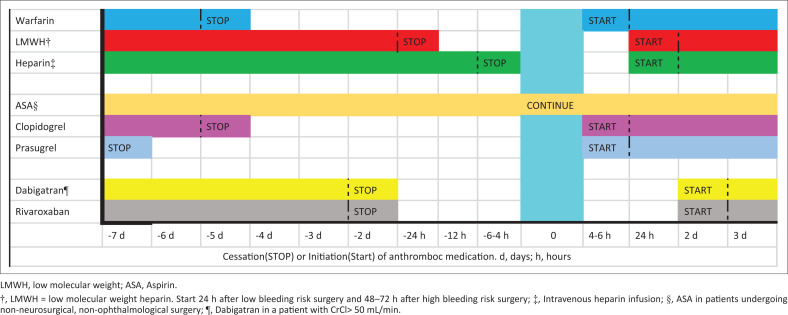
Perioperative management of common anticoagulants in high bleeding risk surgery.

Low molecular weight heparin (1 mg/kg 12 hourly) should be stopped 24 h prior to surgery. Treatment can be recommenced 24 h after low bleeding risk surgery and 48–72 h after high bleeding risk surgery. Intravenous (IV) UFH should be stopped 4–6 h prior to surgery and can be recommenced 24 h after surgery (depending on postoperative bleeding risk). Figure 2 summarises antithrombotic therapy recommendations in high-risk surgery.

### Neuraxial and deep nerve block considerations

**FIGURE 3:**
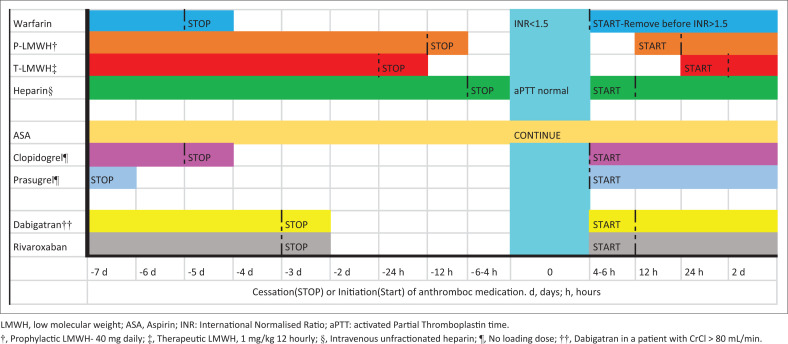
Perioperative management of common anticoagulants during neuraxial anaesthesia.

The most feared complication after neuraxial anaesthesia (spinal and epidural anaesthesia) is a spinal haematoma leading to transient or permanent loss of function. The incidence of significant complications after neuraxial anaesthesia (spinal haematoma and abscess) ranges from 1:6000 cases in non-obstetric anaesthesia to 1:154 000 in obstetric anaesthesia, with epidural anaesthesia carrying a greater risk than spinal anaesthesia.^24^ Spinal haematoma formation carries high morbidity; hence, specific recommendations are made regarding anticoagulants and neuraxial procedures or therapies (Figure 3).^8^ The American Society of Regional Anesthesia and Pain Medicine gives guidance on three clinical situations: how to manage antithrombotic medication prior to neuraxial anaesthesia, how to manage antithrombotic medication after neuraxial anaesthesia and how to manage antithrombotic medication with an epidural catheter in situ.^8^

The European Society of Regional Anaesthesia considers deep nerve blocks to have a high risk of bleeding. The same recommendations apply to neuraxial anaesthesia and deep nerve blocks.^18^ Examples of commonly used deep nerve blocks are deep cervical plexus block, infraclavicular brachial plexus nerve block, thoracic and lumbar paravertebral block, lumbar plexus block, proximal sciatic nerve block and pericapsular nerve block.^18^ The superficial nerve blocks can generally be performed without cessation of anticoagulation.^18^

### Vitamin K antagonists

Neuraxial anaesthesia should only be performed if the INR is less than 1.5. Vitamin K antagonists can be restarted with an epidural catheter in situ but it should only be removed if the INR is less than 1.5.^8^

### Unfractionated heparin

Subcutaneous use of UFH has largely been superceded by LMWH in clinical practice. For this review, IV use of UFH will be discussed.

Unfractionated heparin must be stopped 4–6 h prior to spinal anaesthesia or epidural catheter insertion. The activated partial thromboplastin time (aPTT) must also be normal. Intravenous UFH can be reinitiated within 1 h after spinal anaesthetic or epidural catheter removal. Maintaining an indwelling neuraxial catheter while a patient is on IV UFH is not advised.

In the event of a traumatic neuraxial anaesthetic, it is reasonable to withhold initiation of UFH. However, the guidelines regarding how long to wait before the first postoperative dose after a traumatic neuraxial anaesthetic use is unclear.^8^

### Low molecular weight

Therapeutic LMWH (1 mg/kg, 12 hourly), should be stopped 24 h before neuraxial anaesthesia and can be reinitiated 24 h after catheter removal. In high-bleeding risk surgery, LMWH should only be administered 48–72 h after removal. Therapeutic dosages should not be used with a catheter in situ.^8^ If a decision is made to initiate therapeutic LMWH postoperatively, the indwelling catheter should be removed at least 24 h after it was inserted and therapeutic dosing can be given 4 h after it has been removed.

Neuraxial anaesthesia can be administered 12 h after prophylactic LMWH (0.5 mg/kg daily or 40 mg daily). Daily dosing prophylaxis can be reinitiated 12 h after catheter removal.^8^ In contrast to therapeutic LMWH, epidural catheters can be maintained in situ with once-daily prophylactic dosing. Twelve hours should elapse between the last dose of prophylactic LMWH and catheter removal, and the next prophylactic dose can be given 4 h after catheter removal.

### Direct-acting oral anticoagulants

Factor Xa inhibitors must be stopped 3 days prior to neuraxial anaesthesia and can be restarted 6 h after the procedure. Dabigatran in patients with a CrCl > 80 mL/min must be stopped 3 days prior to neuraxial anaesthesia, similar to the factor Xa inhibitors. Patients with a CrCl of 50 mL/min – 79 mL/min require cessation of medication 4 days before the procedure, and those with a CrCl of 30 mL/min – 49 mL/min should have medication stopped 5 days prior. It is best to avoid neuraxial anaesthesia in individuals with a CrCl<30 mL/min as the duration of action of dabigatran is unpredictable. All DOACs can be restarted 6 h after catheter removal or spinal insertion. Epidural catheters should not be maintained in situ while a patient is on a DOAC.^8^

## Antiplatelet agents

Asprin can be safely continued during neuraxial anaesthesia and requires no dose adjustment.

The intervals from the cessation of medication to neuraxial anaesthesia placement are the same as described above for elective surgery. If no loading dose is used, P2Y12 inhibitors can be initiated immediately post-neuraxial procedure. If a loading dose is needed, 6 h should pass between the procedure and administering of medication. Neuraxial catheters should not be maintained in situ after antiplatelet medication has been given (except ASA).^8^

Figure 3 summarises the recommendations when performing neuraxial anaesthesia.

## Conclusion

The perioperative management of antithrombotic medication is complex and fraught with risk if inappropriate cessation or initiation of treatment is prescribed. However, by adhering to basic pharmacological principles and best practice guidelines, the risks involved can be mitigated, and patient outcomes can be improved.
